# Enhanced delignification of steam-pretreated poplar by a bacterial laccase

**DOI:** 10.1038/srep42121

**Published:** 2017-02-07

**Authors:** Rahul Singh, Jinguang Hu, Matthew R. Regner, James W. Round, John Ralph, John N. Saddler, Lindsay D. Eltis

**Affiliations:** 1 Department of Microbiology & Immunology, The University of British Columbia, Vancouver, BC, V6T 1Z3 Canada; 2 Forest Products Biotechnology/Bioenergy Group, Faculty of Forestry, The University of British Columbia, 2424 Main Mall, Vancouver, BC, V6T 1Z4, Canada; 3 US Department of Energy Great Lakes Bioenergy Research Center, Wisconsin Energy Institute, Madison, WI 53726, USA; 4 Department of Biochemistry, University of Wisconsin, Madison, WI 53706, USA; 5 Department of Biochemistry and Molecular Biology, The University of British Columbia, Vancouver, BC, V6T 1Z3, Canada.

## Abstract

The recalcitrance of woody biomass, particularly its lignin component, hinders its sustainable transformation to fuels and biomaterials. Although the recent discovery of several bacterial ligninases promises the development of novel biocatalysts, these enzymes have largely been characterized using model substrates: direct evidence for their action on biomass is lacking. Herein, we report the delignification of woody biomass by a small laccase (sLac) from *Amycolatopsis sp*. 75iv3. Incubation of steam-pretreated poplar (SPP) with sLac enhanced the release of acid-precipitable polymeric lignin (APPL) by ~6-fold, and reduced the amount of acid-soluble lignin by ~15%. NMR spectrometry revealed that the APPL was significantly syringyl-enriched relative to the original material (~16:1 vs. ~3:1), and that sLac preferentially oxidized syringyl units and altered interunit linkage distributions. sLac’s substrate preference among monoaryls was also consistent with this observation. In addition, sLac treatment reduced the molar mass of the APPL by over 50%, as determined by gel-permeation chromatography coupled with multi-angle light scattering. Finally, sLac acted synergistically with a commercial cellulase cocktail to increase glucose production from SPP ~8%. Overall, this study establishes the lignolytic activity of sLac on woody biomass and highlights the biocatalytic potential of bacterial enzymes.

Lignocellulosic biomass represents the most abundant carbon-based source of energy on earth. Valorization of its three major components – cellulose, hemicelluloses and lignin – is critical for the sustainability of next-generation bio-refineries. The production of fuels, biomaterials and other value-added products from biomass will lower our dependence on fossil fuels and reduce greenhouse gas emissions^1^. Woody biomass (*e.g*., poplar and pine), agriculture residues (*e.g*., corn stover), and perennial grasses (*e.g*., miscanthus and switchgrass) have emerged as the feedstocks of choice based on socio-economic analyses, in part because they do not compete with the food crops^2^,^3^. However, the cost-effective deconstruction of biomass remains a significant barrier to its utilization. This recalcitrance is due principally to the heterogeneity of lignin and its complex association with polysaccharides.

Several biochemical and thermochemical methods have been developed to efficiently deconstruct biomass. Thermochemical conversion, attractive for its low residence times and continuous processing of diverse feedstock, is not optimal for targeting specific end products^1^. In contrast, biochemical methods, which use low-severity thermochemical treatment (pretreatment; 100–200 °C) followed by enzymatic hydrolysis, are better at converting feedstocks to specific end products^1^,^4^. Nevertheless, for efficient digestion and maximum yield, lignin must be dissociated from carbohydrates under milder conditions^4^,^5^. Moreover, inhibition of cellulolytic enzymes by lignin present in woody biomass, such as steam-pretreated poplar (SPP), further reduces the efficiency of the bioconversion^6^. Enzymes able to delignify woody biomass under mild conditions are thus of particular interest.

The majority of lignin-depolymerizing enzymes, or ligninases, are sourced from fungi, whose role in biomass degradation is well documented^7^. The best-characterized of these are multi-copper oxidases, such as laccases (EC 1.10.3.2), and heme-containing peroxidases (EC 1.11.1.7), such as lignin (LiP) and manganese (MnP) peroxidases. LiP is considered to be the most efficient ligninase due to its high reduction potential, which enables it to oxidize non-phenolic lignin. However, laccases are more suitable as biocatalysts because they utilize O_2_ instead of H_2_O_2_ to catalyze substrate oxidation^8^,^9^ and are not prone to cofactor degradation. Recent studies have also established that some bacterial enzymes are able to degrade lignin^10^,^11^,^12^,^13^. Bacterial lignin-degrading enzymes include dye-decolorizing peroxidases (DyPs) and small laccases (sLacs). Genomic and metagenomic approaches have also revealed several laccases of prokaryotic origin^14^. Very recently, a thermostable variant of a bacterial multicopper oxidase, CotA, was patented for its ability to improve the efficiency of thermomechanical pulping^15^. Despite this potential, the role of bacteria in depolymerizing lignin remains unclear; activities have been mostly established using model substrates or isolated lignin preparations, with limited studies using native lignin or lignocellulosic biomass^10^,^11^,^12^,^13^,^16^,^17^,^18^,^19^,^20^,^21^,^22^.

Laccases have a cupredoxin fold that binds four copper ions arranged in a mononuclear and a trinuclear cluster^23^. Substrate oxidation at the mononuclear site generates electrons that are transferred to the trinuclear site where O_2_ is reduced to H_2_O. Laccases oxidize a variety of phenolic and non-phenolic substrates, and their catalytic efficiency is usually enhanced by mediators, such as ABTS and TEMPO^24^. Accordingly, the role of fungal laccase and laccase-mediator systems in delignification of lignocellulose, particularly Kraft wood pulp is well established^25^. A hallmark of the bacterial sLacs is that they lack one of the three domains of the fungal laccases. Nevertheless, the two-domain sLac has the cupredoxin fold and the two metallocentres of the larger homologs^14^. The sLacs characterized to date have a lower reduction potential than the fungal laccases (~375 mV *vs*. up to 800 mV)^26^, but genetic evidence suggests that sLac contributes to the delignification of milled miscanthus by *Streptomyces coelicolor*^22^. The reported stability of sLac across a wide pH and temperature together with the relative ease of production and engineering of bacterial enzymes makes sLac an attractive biocatalyst.

Herein, we report the delignification of woody biomass by sLac from *Amycolatopsis sp*. 75iv2. Of a variety of biomass samples pretreated with steam or solvent, sLac delignified SPP the most efficiently. The resulting lignin was isolated and characterized using GPC-MALS and NMR spectrometry. Lignin-derived compounds produced or depleted during sLac treatment were also identified using GC-MS, and the preference of sLac for a range of lignin-derived monoaryls was determined. Finally, the treated biomass was subjected to enzymatic hydrolysis to evaluate the effect of sLac on sugar production. Our study provides evidence for delignification and lignin depolymerization by a bacterial enzyme and provides insight into how this occurs.

## Results and Discussion

### Production and purification of sLac

The sLac from *Amycolatopsis sp*. 75iv2 was recently characterized as a lignin-degrading enzyme^22^. Intriguingly, this is the second lignin-degrading enzyme characterized from this bacterium, the first being a DyP-type peroxidase^17^. We focused on the sLac to investigate the biocatalytic potential of these enzymes. We began by modifying the purification process to maximize the incorporation of copper ions and the enzyme’s specific activity. More particularly, the addition of 0.2 mM CuSO_4_ to the culture medium prior to the induction of sLac production and the addition of 5 mM CuSO_4_ to the lysis buffer significantly improved the metal incorporation and yielded ~20 mg sLac per litre of culture. The absorption spectrum of the purified sLac was typical of those for blue copper oxidases with maxima at 280 and 595 nm (A_280/595_ ~12) and a shoulder at 330 nm (Fig. S1). ICP-MS analyses revealed 4.2 ± 0.2 Cu ions per molecule of sLac. Steady state kinetic analyses (Fig. S2) established that the enzyme was catalytically active over a range of pH, oxidizing ABTS at pH 5 (*k*_cat_/*K*_m_ 14 ± 0.5 mM^−1^s^−1^) and syringol (2,6-dimethoxyphenol) at pH 8 (*k*_cat_/*K*_m_ 0.17 ± 0.04 mM^−1^s^−1^). These spectroscopic and kinetic properties are comparable to those reported for sLac from *Streptomyces veridochromogenes* (sLac_SvSL_)^27^.

### Incubation of sLac with different pretreated biomass

**Figure 1:**
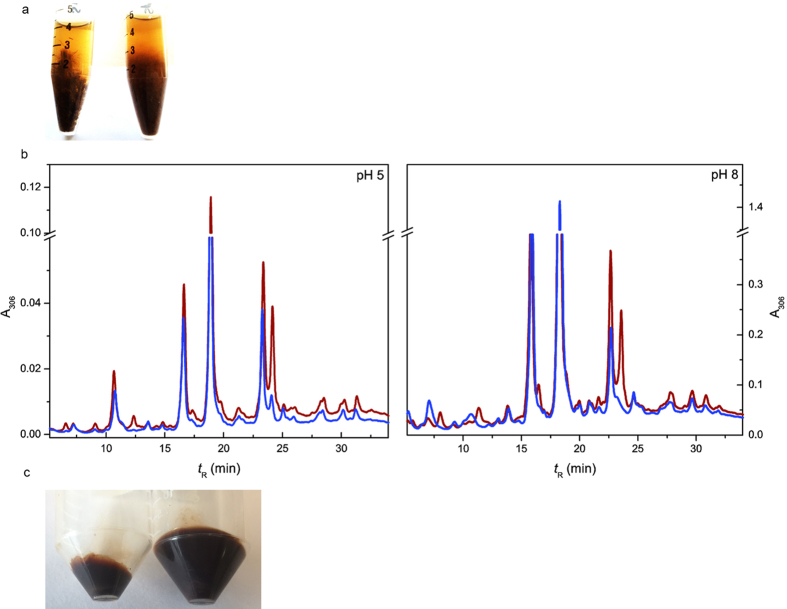
Extraction of lignin from SPP. (**a**) Reaction mixtures containing SPP suspended in 50 mM Tris-HCl, pH 8.0, without (left) or with (right) 0.5 μM sLac. (**b**) HPLC analyses of the soluble portion of the reaction mixtures at pH 5 and 8. Elution profile of the samples incubated without (brown) or with sLac (blue) is shown. (**c**) APPL recovered from SPP incubated without (left) or with (right) sLac.

To evaluate the effect of sLac on heterogeneous lignocellulosic feedstocks as produced and used by biorefineries, we investigated the action of the enzyme on steam- or organosolv-pretreated biomass from poplar, lodgepole pine, and corn stover. These substrates were used without further processing, like kraft pulping^28^ or milling to reduce the particle size, a common practice to provide better substrate accessibility^22^. Biomass, varying in fiber size from a few millimeters to over a centimeter, was suspended in buffered aqueous solutions (pH 8) and incubated with or without sLac (Fig. 1a) for 48 h at 30 °C. The biomass loading (2% w/v) was based on what is routinely used for hydrolysis using cellulases^29^. HPLC analysis of the incubated samples revealed that for all four types of biomass, sLac resulted in the depletion of a number of peaks in the chromatograms of the soluble fractions (Fig. S3). However, the effect of sLac on SPP was notable for the appearance of new peaks in the chromatogram. The same phenomenon was observed with SPP whether the reaction was performed at pH 5 or 8 (Fig. 1b).

### Identification of lignin-derived compounds

**Figure 2:**
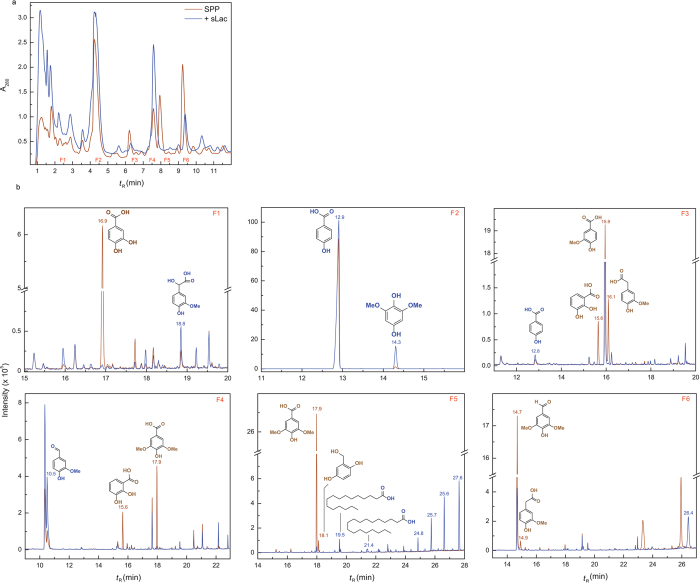
Identification of compounds produced or depleted during incubation of SPP with sLac. (**a**) The HPLC elution profile of the soluble portion of the reactions containing SPP with or without sLac. (**b**) The GC traces of the TMS-derivatized samples of HPLC fractions F1-6. The shown structures are those of the best hits obtained from the reference library or confirmed using standards on HPLC as noted in Table 1.

To identify monoaryls produced during SPP incubation, soluble fractions obtained from 50 mL reactions were concentrated using solid-phase extraction (SPE)^18^ and fractionated using HPLC (Fig. 2, top). GC-MS analyses of the TMS-derivatized fractions (Fig. 2) revealed that, as compared to control reactions, sLac treatment resulted in the depletion of phenolic acids, such as syringate, vanillate and protocatechuate, as well as syringaldehyde (Table 1). sLac treatment also resulted in an ~3-fold increase in vanillin levels together with increased levels of 4-hydroxybenzoate and 2,6-dimethoxybenzene-1,4-diol.

**Figure 3:**
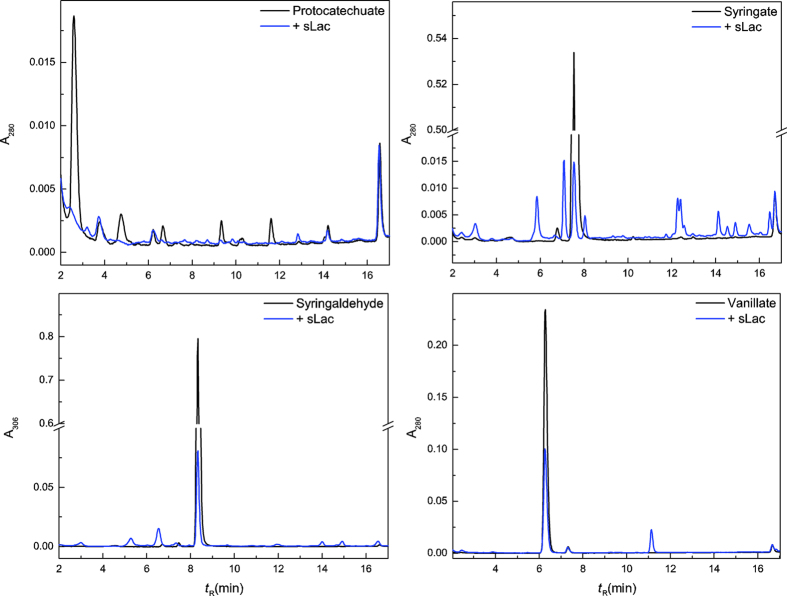
HPLC analyses of sLac-catalyzed oxidation of phenolic acids and aldehydes. Reaction mixtures containing 100 μM of either protocatechuate, syringate, syringaldehyde or vanillate were incubated in 50 mM Tris, pH 8 with or without 0.5 μM sLac for 48 h at 30 °C. The samples were acidified prior to HPLC analysis.

We investigated whether these compounds were substrates for sLac by incubating 100 μM of each of them with the enzyme under the conditions used to incubate SPP. HPLC and GC-MS analyses (Fig. 3 and S4) revealed that sLac catalyzed essentially the complete oxidation of syringate, protocatechuate, and syringaldehyde over 48 h at 30 °C, while vanillate was oxidized less efficiently. Moreover, sLac oxidized just over half of the syringate and syringol over 2 h, while vanillate was not detectably oxidized (Fig. S5). Finally, syringate oxidation yielded multiple HPLC-detectable products (Fig. S6). Attempts to identify these products using GC-MS were unsuccessful.

These data are consistent with those reported for fungal laccases and reflect the midpoint reduction potentials of the monoaryls. For example, a laccase from *Rhizoctonia praticola* oxidized syringate and syringol at similar rates while that of vanillate was ~7-fold lower^30^. Similarly, fungal laccases preferentially oxidize some phenolic aldehydes, such as syringaldehyde, over others, such as vanillin^31^. Efficiently oxidized monoaryls, such as protocatechuate and syringate, have potentials between ~0.4–0.5 V^32^, while those oxidized less efficiently, such as vanillate, have higher potentials (~0.73 V)^32^. Interestingly, only a few reports of the oxidation of protocatechuate by fungal laccases exist^33^. Although protocatechuate likely undergoes auto-oxidation, sLac clearly accelerated its depletion (Fig. 3, top left; Fig. S4, top). Finally, sLac’s inability to oxidize vanillin likely contributes to this compound’s enrichment in the soluble fraction of enzyme-treated SPP.

### Acid-precipitable polymeric lignin (APPL) from SPP – yield and size

Incubations of SPP were further distinguished by the production of APPL, observed upon acidification of the soluble fraction: incubation of the other biomass samples, including organosolv pretreated polar, did not yield any detectable APPL. The yield of APPL was maximal after 48 h incubation, whereas short-term incubations (2–4 h), as reported for the treatment of kraft pulp by fungal enzymes^25^, did not produce appreciable APPL. Although some APPL was produced from SPP in the absence of sLac, the yield was ~6-fold higher in its presence (Fig. 1c). Moreover, the yield of APPL was dependent on pH: none was observed at pH 5, although the HPLC analyses clearly established that sLac modified the lignin at this pH (Fig. 1b, left). Finally, APPL production also depended on the concentrations of sLac and SPP. Thus, lowering the sLac concentration or increasing that of SPP above 2% w/v, lowered the yield of APPL.

To investigate whether the sLac-catalyzed oxidation of monoaryls present in the aqueous suspension of SPP contributed towards APPL production, the soluble portion of the SPP was incubated with sLac for 48 h. However, no increase in the yield of APPL was observed. Similarly, incubation of syringate and syringaldehyde with sLac at pH 8 also failed to produce any APPL, although substantial precipitate formed over the course of the reaction. Overall, these results indicate that the high yield of APPL in the enzyme-treated sample was due to the action of sLac on the biomass. We estimate that sLac released up to ~10% of the total lignin in SPP based on the latter’s lignin content (~30%) as previously determined^34^. A Klason analysis revealed that SPP contained comparable amounts of APPL whether it was incubated with or without sLac (Table S1). However, SPP contained ~15% less acid-soluble lignin after sLac treatment. Although, it is possible that sLac catalyzed polymerization of phenolics, such as syringate and syringaldehyde^35^, may have contributed to the Klason analysis, the extent of delignification by sLac was relatively modest. Although a fungal laccase-mediator systems achieved up to 50% delignification of eucalyptus biomass^36^, this required multiple cycles of enzyme treatment.

**Figure 4:**
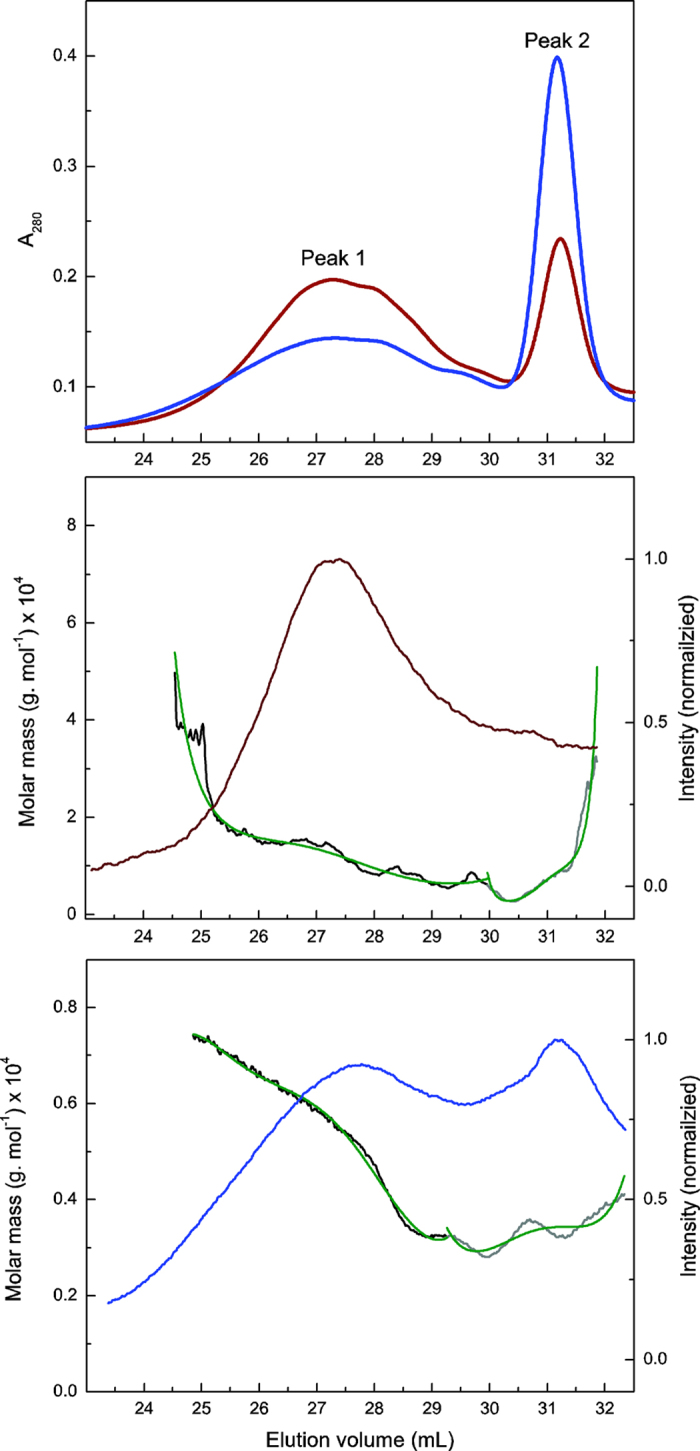
GPC-MALS analysis of APPL extracted from SPP incubated without (brown) or with sLac (blue). The top panel shows the response from the multi-wavelength detector (280 nm). The lower panels show the response of the MALS detector to APPL isolated from SPP without (middle) and with (bottom) sLac. The green curves represent multiple exponential fits of the molar mass distribution to the MALS data obtained from the areas under Peaks 1 (black) and 2 (grey).

**Figure 5:**
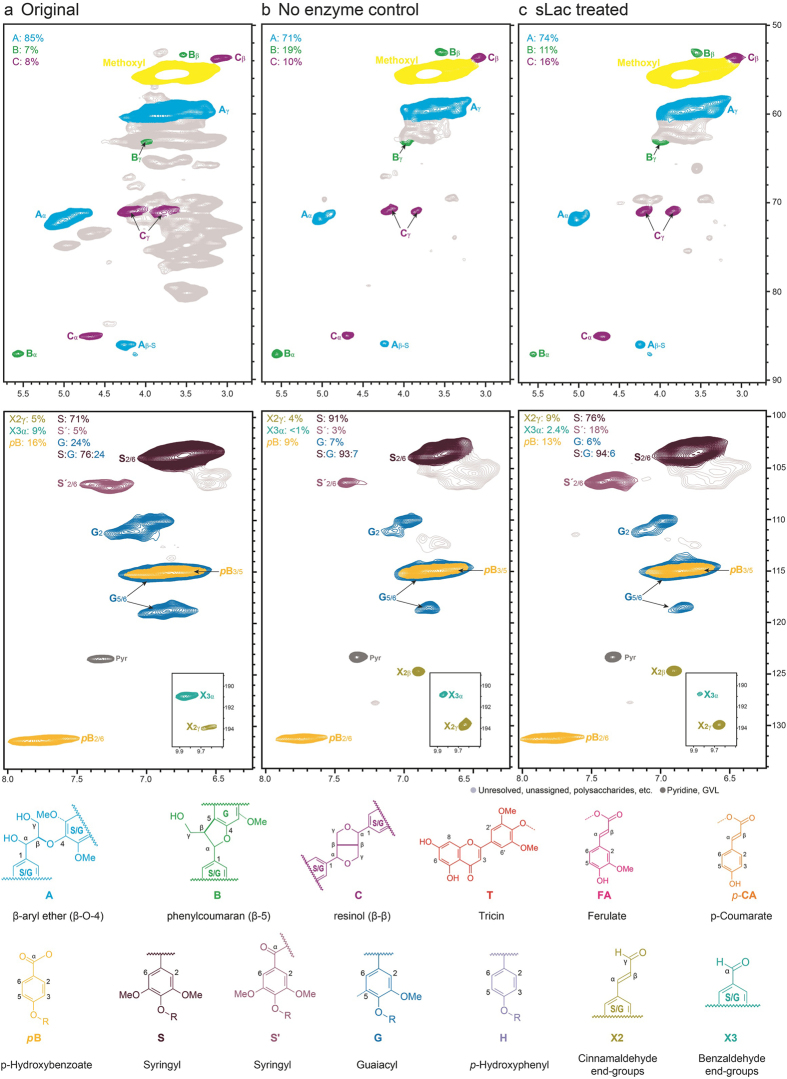
Solution-state ^1^H–^13^C (HSQC) NMR spectra of the SPP biomass (**a**) and APPL obtained from SPP incubated without (**b**) or with (**c**) sLac. The top and the bottom panels show the aliphatic and aromatic regions, respectively, of the 2D-NMR spectra.

To characterize the APPL, it was recovered via centrifugation and purified by dissolving in dioxane and re-precipitating over ice cold HCl (0.1%)^37^. The recovered APPL was acetylated using pyridine and acetic anhydride and characterized using GPC-MALS and NMR spectrometry. GPC analyses established that the APPL from sLac-treated SPP differed significantly from that of the no-enzyme control (Fig. 4, top). More specifically, the APPL recovered using sLac contained less high molecular weight material (Peak 1) and more low molecular weight material (Peak 2) than that recovered without enzyme. This result is consistent with sLac’s catalyzing the depolymerization of lignin^38^. To determine the absolute molar mass, the MALS data, collected using a detector fitted with interference filters to remove the fluorescence from lignin, was analyzed using multi-exponential equations (Fig. 4, lower panels). The results revealed that the weight-average molar masses (*M*_w_) of Peaks 1 and 2 were 45 and 55% lower, respectively, in the sLac-treated APPL as compared to the control sample (Table 2). Similarly, the number-average molar mass (*M*_*n*_) for Peaks 1 and 2 were 49% and 21% lower, respectively, in the sLac-treated APPL. Finally, the polydispersity (*M*_*w*_/*M*_*n*_) of the two peaks was reduced 20–40% after the enzyme treatment. In performing these analyses, the lignin was handled to minimize interaction with the GPC matrix (*i.e*., acetylated, stored for 2 days prior to injection). Importantly, the NMR data (Fig. 5) clearly establish that two APPL samples (liberated with and without sLac) have similar functionalities (*e.g*., very similar S:G ratios), and thus are likely to interact similarly with the column matrix.

Overall, the GPC-MALS data indicate that sLac-treated SPP yielded depolymerized lignin relative to the no enzyme control, a conclusion further supported by the detection of monoaryls by HPLC and GC-MS, and that enzyme treatment lowers polydispersity of lignin. It is likely that sLac selectively depolymerizes the most surface-accessible lignin, although delignification of less accessible lignin might also occur through the action of mediators, such as syringate, vanillate, protocatechuate and syringaldehyde^31^, which are present in SPP and are substrates for sLac (Fig. 3). As other laccase mediators, such as ABTS and TEMPO^24^, are most effective at low pH, these are not useful for sLac-mediated delignification at pH 8; however; ongoing studies focused on identification and optimization of sLac-mediator systems are expected to provide better insight. Further studies are required to investigate the role of these and other mediators in the sLac-catalyzed delignification of SPP.

### NMR analyses of APPL from SPP

NMR analyses of the original SPP and the APPL recovered with and without sLac provided insight into how the enzyme modified the lignin (Fig. 5). First, the S:G ratio of both APPL samples (~16:1) was higher than in the untreated SPP (~3:1). This suggests that S-lignin is preferentially released during incubation or that G-lignin is preferentially degraded. Consistent with the oxidative activity of sLac, the APPL recovered with the enzyme displayed more benzylic oxidation of S-lignin than that of the no-enzyme control. This mirrors the substrate preference of sLac among monoaryls, discussed above, and supports the hypothesis that sLac plays a role in lignin deconstruction. Moreover, the monoaryls identified as substrates for sLac can function as mediators for this deconstruction, as reported for fungal laccases^24^,^39^. The proportions of cinnamaldehyde and benzaldehyde end-groups and *p*-hydroxy benzoate esters appear to vary widely across the three NMR spectra. Integration of 2D NMR contours in the spectra of lignins or plant whole cell walls tends to overestimate these moieties as a result of the difference in relaxation times between end-groups and units more integral to the polymer, but relative levels are considered to be reasonably accurate^40^. Recent experiments have shown benzylic oxidation taking place after Cα–Cβ cleavage under oxidative conditions, and may account for the increase in (hydroxy)benzaldehydes in the enzyme-treated sample over the control; (hydroxy)cinnamyl alcohol end-groups are vinylogous benzylic alcohols so can oxidize to (hydroxy)cinnamaldehydes by similar mechanisms^41^. Again, however, the integrals of these peaks, by the nature of the NMR experiment, lack the necessary accuracy to draw definite conclusions about those relatively mobile components compared to those relaxing more slowly.

Examination of the aliphatic region for each sample offers another perspective on the changes engendered in the lignin by sLac activity. For instance, resinol structures **C** occur at higher levels in the enzyme-treated APPL, an S-rich lignin sample, concordant with previous results suggesting that S-lignin contains resinol structures in greater abundance than G-lignin. Interestingly, however, the control APPL sample, which still exhibits a much higher S:G ratio than average, contains a similar proportion of resinol structures compared to the original SPP biomass. The control APPL sample also shows markedly higher levels of phenylcoumarans **B**, whereas the enzyme-treated APPL sample mostly resembles the original SPP one. Perhaps incubation in the buffered solution (pH 8) selectively releases phenylcoumarans, which may then be oxidatively degraded by sLac. The enzyme-treated sample also contained a small but significant increase in the level of benzylic-oxidized β-ether units (not seen in Fig. 5c, but visible when plotted at lower contour levels); these coincide with the peaks from **A**” in Fig. 3b of Rahimi *et al*.^42^. The low level suggests that cleavage of the chains (to produce the above-noted aldehydes and acids) occurs relatively easily under these conditions after such oxidations. Whatever the cause, the large increase in recovered lignin from the enzyme-treated sample in conjunction with an elevated S:G ratio (and concomitant benzylic oxidation) as demonstrated by NMR suggests that this method could provide access to significant quantities of useful, isolated lignin. Furthermore, evidence of sLac lignolytic activity offers new insight into the oxidative degradation of lignin.

### Synergistic actions of sLac and cellulases

**Figure 6:**
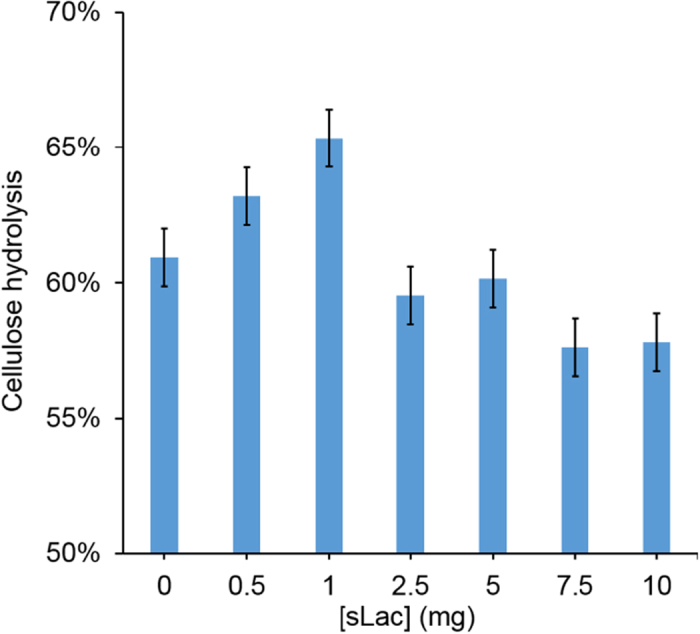
Synergy between sLac and Celluclast^®^ 1.5 L during hydrolysis of the SPP. The data shows the extent of cellulose hydrolysis after 72 h using different dosages of protein (mg/g cellulose). Reactions were incubated in a rotary shaker for 72 h at 30 °C in 50 mM sodium acetate, pH 5. Reactions were performed in triplicate.

To assess the possible synergistic interaction of sLac and cellulases for improved hydrolysis of the biomass, different amounts of sLac (from 0.5 to 10 mg/g cellulose) were added to reaction mixtures containing SPP and Celluclast^®^ 1.5 L, a commercial cocktail of cellulases. A cellulase loading of 10 mg g^−1^ cellulose was used to ensure efficient hydrolysis of cellulose present in the SPP within 48 h. At pH 5, the presence of sLac resulted in modest improvement in the hydrolyis in a dose-dependent manner (Fig. 6). The greatest boosting effect, ~8%, was observed at 1 mg of sLac. No synergistic effect was observed when sLac was replaced with a non-lignolytic protein (data not shown). Moreover, sLac showed no synergistic effect in the cellulytic hydrolysis of organosolv pretreated poplar or the other biomass types tested herein. This is consistent with the observation that sLac did not significantly delignify these other biomasses. The synergy observed between sLac and the commercial cellulase cocktail is similar to those reported for cellulases and other accessory proteins, such as hemicelluases, fungal ligninase, cellulose-disrupting proteins, and lytic polysaccharide monooxygenases^29^. The boosting effect of sLac might be due to improved accessibility for Celluclast^®^ 1.5 L to cellulose within the pretreated biomass or due to detoxification/oxidation of inhibitory compounds by the ligninase. Importantly, sLac treatment did not result in the detectable release of APPL from SPP at pH 5 (*vide supra*), indicating the delignification did not significantly contribute to improved sacharification and that the synergy can be further optimized. The slight decrease in the hydrolysis at higher sLac loading (Fig. 6) might be the result of competitive adsorption of sLac onto cellulose^24^. Overall, these results establish for the first time the synergistic interaction between cellulases and a bacterial ligninase. Further efforts are warranted to optimize “cellulase cocktails” for improved and efficient biomass biological deconstruction processes.

### Proposed mechanism of sLac-catalyzed delignification of SPP

**Figure 7:**
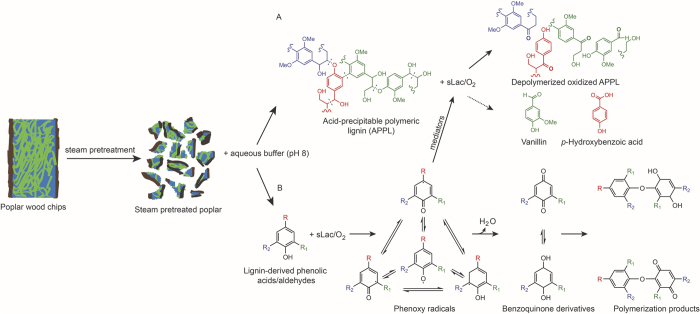
A proposed mechanism for the sLac-catalyzed delignification of SPP. Representations of poplar wood chips and the steam-pretreated fragments show cellulose (blue), hemicelluloses (green) and lignin (brown). Path A shows the release of APPL from SPP followed by depolymerization, benzylic oxidation (bold), and the production of monoaryls due to the direct interaction of sLac with lignin. Path B shows the sLac-mediated oxidation of monoaryls released into the aqueous phase during the incubation of SPP. The generated phenoxy radicals can either act as mediators to initiate radical depolymerization of the lignin or initiate polymerization reactions. For protocatechuic acid R=COOH, R_1_=OH and R_2_=H; for syringic acid R=COOH, R_1_=OCH_3_ and R_2_=OCH_3_; for syringaldehyde R=CHO, R_1_=OCH_3_ and R_2_=OCH_3_; for vanillic acid R=COOH, R_1_=OCH_3_ and R_2_=H.

The results from NMR spectroscopy and GC-MS indicate that sLac catalyzes the depolymerization of lignin using a mechanism similar to that proposed for fungal laccases^23^,^39^ (Fig. 7). Briefly, sLac catalyzes the benzylic oxidation of polymeric lignin leading to the cleavage of intersubunit C-C and C-O bonds. This leads to the depolymerisation of low molecular weight lignin as observed in the GPC-MALS analysis. It is possible that sLac also catalyzes the oxidation of monoaryls present in the SPP to produce phenoxyl radicals. Such radicals can lead to the production of benzoquinone derivatives and polymerization products. Alternatively, these radicals can function as mediators to initiate cleavage of C–C and β-aryl bonds in the lignin^43^,^44^. Further studies are required to identify monoaryls that act as mediators.

## Conclusions

This study provides unambiguous evidence for the delignification and depolymerization of woody biomass by a bacterial enzyme: treatment of SPP with sLac resulted in a ~6-fold increase of APPL, and this APPL was of lower molecular mass than that released in the absence of sLac. Moreover, sLac preferentially oxidized S-type subunits versus G- and H-subunits, both in APPL, as well as in solution (*e.g*., syringate and syringaldehyde versus *p*-hydroxybenzoate and vanillin). This suggests that sLac deconstructs lignin using a mechanism similar to that proposed for fungal laccases^45^. Briefly, sLac catalyzes (A) the benzylic oxidation of polymeric lignin with concomitant cleavage of C-C and C-O bonds, as well as (B) the production of phenoxy radicals, which can either function as mediators or lead to benzoquinone derivatives and polymerization products (Fig. 7). It is unclear to what extent sLac acts directly on lignin as opposed to through the action of phenolics present in SPP serving as mediators. Studies using radiolabeled synthetic lignin and similar approaches would provide better insight into this, as well as the extent of polymerization versus depolymerization. Similarly, structural characterization of sLac to identify substrate-binding pocket(s), such as those for syringate or syringaldehyde, would provide further mechanistic insights and improve the basis for engineering the enzyme for more efficient biomass deconstruction. Thus, the identification of an acetovanillone binding site ~10 Ǻ from the T1 copper site in a sLac homologue *in cristallo*^22^ does not rule out the existence of multiple substrate binding pockets. It would be particularly interesting to determine if sLac binds to lignin residues directly or preferentially uses monoaryls as mediators.

Overall, this study validates the potential of bacterial enzymes as biocatalysts due to their broad substrate specificity and stability: sLac retained ~100% of its activity post incubation with SPP in the current studies. More specifically, sLac-treatment of SPP represents a relatively simple method to isolate lignin for research and biotechnological applications, such as production of fiberboards and carbon fibers^46^,^47^. The study also demonstrates that sLac acts synergistically with a commercial cocktail of cellulases, improving hydrolysis of the SPP biomass. Although the boosting effect was modest as compared to that reported for fungal laccases^48^, this is the first such report for a bacterial ligninase and further optimization should be possible. Further studies on sLac and other bacterial ligninases, particularly in combination with natural and artificial mediators, to enhance reduction potential, will hasten the development of bacterial biocatalysts for efficient biomass deconstruction and lignin valorization.

## Methods

All reagents and biochemicals were purchased from SIGMA-Aldrich, ACROS, or Fisher and were used without further purification. Data analysis and figures were prepared using Origin 8.1 (Origin Lab Corporation) and Adobe Illustrator CS5.1.

### DNA Manipulation and Plasmid Construction

The portion of sLac corresponding to the product without the putative N-terminal TAT signal sequence was amplified using the genomic DNA isolated from *Amycolatopsis* sp 75iv2 as template. The polymerase chain reactions (PCR) were carried out using the Expand High-Fidelity Polymerase (Roche) on the Veriti 96-well Thermal Cycler (Applied Bio-systems). The gene was cloned into pET15a(+) (Novagen) under the control of the T7 promoter utilizing the following primers: 5′-ATCAATTCATATGCAGGGCACGACCCGGCGGATC-3′ (*slac* forward) and ATCAATTGGATCCTCAGTGTTCGTGGACACCG (*slac* reverse). The resulting recombinant protein include a TEV protease cleavable N-terminal poly-His tag and residues 6–279 for sLac. All primers were synthesized and final clones sequenced at the Nucleic Acid-Protein Services Unit (The University of British Columbia).

### Bacterial Strains and Growth

His-tagged sLac was overexpressed in *E. coli* Rosetta™ BL21(DE3)pLysS. The cells harboring pET15a(+)_sLac were grown in LB containing 100 μg/mL of ampicillin (MP biomedicals) by incubating at 37 °C with shaking (200 rpm). Gene expression was induced with 1 mM isopropyl-β-D-thiogalactopyranoside (IPTG) when the cell density reached an A_600_ of 0.5. At this time, 0.2 mM CuSO_4_ was also added to the growth medium and cells were incubated at 25 °C for a further 16 h with shaking. Post incubation, the cells were harvested by centrifugation and the pellet was washed twice with 50 mM Tris-HCl, pH 8.0 (Buffer A) and frozen at –80 °C until use.

### Protein Purification

All protein purification work was conducted at 4 °C unless otherwise stated. Frozen cell pellet was thawed and the cells were resuspended in Buffer A containing 5 mM CuSO_4_, protease inhibitor (5 mg/mL) (Sigma Catalog no. S8830) and DNAse (2 μg/mL). The cell suspension was passed (5×) through an Emulsi Flex-C5 homogenizer (Avestin) and the lysed cells were centrifuged at 30,500× *g* for 50 min. The soluble portion was filtered (0.45 μm) and incubated with Ni-Sepharose (6 Fast Flow, GE) resin for 1 h before packing on to a glass column for purification. After collecting the eluate, the protein bound resin was washed with 25 column volumes (CV) of buffer A followed by a second wash using 4 CV of buffer A containing 20 mM imidazole. Finally, the protein was eluted using 5 CV of buffer A with 0.5 M imidazole. The eluate was concentrated, using a 30 kDa MWCO membrane Amicon Ultra-15 Centrifugal Filter Device (Millipore), to ~2 mL and dialyzed against 6 L of buffer A. Post dialysis the protein sample was centrifuged (10 min, 25,000× *g*) and aliquots were flash frozen in liquid nitrogen and stored at –80 °C.

The molecular weight and purity of the protein (>95% apparent homogeneity) were confirmed using SDS-PAGE. Protein concentration was determined using ε_280_ 54,890 M^−1 ^cm^−1^ (based on the truncated amino acid sequence lacking the signal peptide), and the presence of Cu was confirmed by using ICP-MS (UBC). For routine analysis, ε_595_ 4400 M^−1 ^cm^−1^ was used to determine Cu^2+^ concentration^49^, and the ratio of A_280/595_ was used to compare Cu^2+^ incorporation in multiple preparations of sLac.

### Steady-state kinetic parameters

Enzyme assays were performed in triplicate in 1 mL at 30 °C using a Cary 5000 spectrophotometer fitted with a thermostatted cuvette holder. The steady-state kinetics parameters for ABTS oxidation were obtained by monitoring the change in absorbance at 414 nm (ε 36.6 mM^−1 ^cm^−1^) in reaction mixtures containing 0.01–10 mM ABTS, 0.1 μM sLac, and 50 mM sodium acetate at pH 5. Those for syringol were obtained by monitoring the change in absorbance at 468 nm (ε = 37.5 mM^−1 ^cm^−1^) in reaction mixtures containing 0.05–10 mM syringol, 0.1 μM sLac, and 50 mM Tris-HCL (pH 8).

### Preparation of steam- and organosolv-pretreated biomass

Steam- and organosolv-pretreated corn stover, poplar, and lodgepole pine were produced in the Forest Products Biotechnology/Bioenergy Laboratory as described previously^50^. The chemical composition of each substrate’s water insoluble fraction after pretreatment was determined using the modified Klason lignin method derived from the TAPPI standard method T222 om-88 as previously described^29^.

### Incubation of steam- or organosolv-pretreated biomass with sLac

Biomass samples (2% w/v) were incubated at 30 °C and 200 rpm for 48 h with or without 0.5 μM sLac in 5, 50 or 2000 mL 50 mM Tris-HCL, pH 8. Following incubation, the insoluble portion of the biomass was removed via centrifugation (3100× *g*), while the soluble portion was filtered through a 0.45 μm membrane filter (Whatman) and acidified, using HCl (10% final concentration). Precipitated enzyme and lignin (APPL) upon acidification was collected via centrifugation (3100× *g*). APPL was further purified by dissolving in dioxane (98%) followed by passage through a 0.45μm membrane filter; the lignin was precipitated in 100 mL ice-cold 0.1% HCl. Finally, the precipitated purified APPL was recovered by centrifugation and freeze dried before storing in the dark at ambient temperature. Acid-precipitable and -soluble lignin content of SPP were determined by Klason lignin analysis^44^.

#### Oxidation of phenolic acids and aldehydes by sLac

Reactions containing 100 μM substrate (protocatechuate, syringate, syringaldehyde or vanillate) were incubated at 30 °C for 48 h with or without 0.5 μM sLac (50 mM Tris-HCl, pH 8.0). Post incubation the samples were analyzed using HPLC and GC-MS as described below. For comparison with syringol, the substrate concentration was increased to 200 μM and the incubation time was reduced to 2 h.

### Characterization of the soluble fractions from the reactions

The acidified soluble portions of the reaction mixtures were initially analyzed on HPLC using a Waters 2695 separations module equipped with a Waters 2996 photodiode array detector. Filtered (0.2 μm) samples (100 μL) were injected onto a 150 mm × 3.00 mm C_18_ column (100 Å, 5 μ; Phenomenex, Torrance, CA) operated at a flow rate of 0.7 mL/min and equilibrated with aqueous 0.5% formic acid, 10% methanol. Reaction products were eluted using the following gradient: 10% MeOH for 2 min, 10 to 50% MeOH over 28 min, 50 to 70% MeOH over 5 min, and 100% MeOH over 3 min. To identify the compounds in the soluble portion of the SPP reactions, the compounds were first concentrated using solid-phase extraction^18^ and then fractionated using a 150 mm × 3.00 mm Kinetex EVO C_18_ column (100 Å, 5 μ; Phenomenex, Torrance, CA) and the following gradient: 10% MeOH for 2 min, 10 to 50% MeOH over 13 min, 50 to 100% MeOH over 1 min, and 100% MeOH over 6 min. Fractions collected from the HPLC were extracted using ethyl acetate, air dried, dissolved in pyridine, and derivatized using BSTFA + TMCS- (99:1). Derivatized fractions were analyzed by GC-MS using an HP 6890 series GC system fitted with an HP 5973 mass-selective detector and a 30 m × 250 μm HP-5MS Agilent column. The operating conditions were: *T*_GC_ (injector), 280 °C; *T*_MS_ (ion source), 230 °C; oven time program (*T*_0 min_), 120 °C; *T*_2 min_, 120 °C; *T*_30 min_, 260 °C (heating rate 5 °C·min^−1^); and *T*_37 min_, 260 °C.

### Gel Permeation Chromatography coupled multi-angle light scattering (GPC-MALS) of APPL

GPC-MALS was used to determine the absolute molar mass distribution of APPL recovered from SPP. The reaction was scaled up (1 L) to produce up to 60 mg of APPL. The samples containing 20 mg of freeze dried APPL were acetylated in a mixture (1:1) of pyridine and acetic anhydride at RT for 24 h. Acetylated APPL was recovered following precipitation in ice cold acidic water (0.1% HCl) and freeze dried. Subsequently, the acetylated samples (5 mg) were dissolved in dry tetrahydrofuran (THF) amended with butylated hydroxytoluene (BHT) as stabilizer (Sigma) and stored for 2 days prior to injection to minimize its interactions with the GPC matrix^51^. GPC was conducted using an Agilent 1200 series HPLC equipped with a multi-angle wavelength detector, downstream of which were MALS, viscosity, and refractive index (RI) detectors (DAWN-HELEOS, Wyatt Technologies, Santa Barbara, CA, USA). To eliminate signal contributions from lignin fluorescence, the laser polarized light of the MALS detector was set at 785 nm, and only even-numbered detectors equipped with interference filters were used for molar mass determination. The samples were filtered (0.2 μm) and injected (100 μL) on Styragel (Waters, Milford, MA, USA) columns HR 4, HR 3, and HR 1 connected in series and were run at 35 °C with THF as the eluting solvent (0.7 mL min^−1^). The MALS detector was normalized using a 30 kDa monodisperse polystyrene standard. The molar mass of the APPL samples was determined using multi-exponential equations to fit the MALS data using Astra^®^ 6 (Wyatt Technologies, Santa Barbara, CA, USA).

### NMR analysis of SPP and APPL

Dried SPP biomass samples were placed into two stainless steel screw-top grinding jars (50 mL) along with six stainless steel ball bearings (10 mm) and pre-ground for 3 min at 30 Hz in a Retsch (Newton, PA, USA) Mixer Mill MM 400. These pre-ground cell walls were then extracted via sonication in a Branson (Danbury, CT, USA) 3510 Ultrasonic Cleaner for 20 min with distilled water (4 × 40 mL), 80% ethanol (4 × 40 mL), and acetone (4 × 40 mL). After spinning the samples down between every extraction in a Thermo Scientific (Pittsburgh, PA, USA) Biofuge Primo Centrifuge for 20 min at 8000 rpm (8873 RGF), the supernatant was discarded. Isolated cell walls were then lyophilized overnight in an SP Scientific (Warminster, PA, USA) VirTis Freezemobile 35ES and finely ground in a Fritsch (Idar-Oberstein, Germany) Planetary Micro Mill Pulverisette 7 in zirconium dioxide (ZrO_2_) vessels (20 mL) containing ten ZrO_2_ ball bearings (10 mm). The material was ground at 600 rpm over 13 grinding cycles of 20 min intervals, with 10 min interval breaks between each cycle. Ball-milled cell walls were recovered from the vessels using distilled water and were again lyophilized overnight. APPL samples recovered from the SPP were analysed by NMR without further modification.

Plant cell wall gel-samples were prepared as described previously^52^. Approximately 80 mg of dry, ball-milled cell walls, or 10–30 mg APPL, were added to 5 mm NMR tubes, followed by the addition of 500 μL DMSO-*d*_*6*_/ pyridine-*d*_*5*_ (4:1). A small neodymium magnet was used to homogenize the resulting viscous mixture, after which each sample was sonicated for approximately 1 h.

NMR spectra were acquired on a Bruker Biospin AVANCE 700 MHz spectrometer fitted with a cryogenically cooled 5-mm QCI (^1^H/^31^P/^13^C/^15^N) gradient probe with inverse geometry (proton coils closest to the sample). The central residual DMSO peak was used as a reference (δ_C_, 39.52 ppm; δ_H_, 2.50 ppm). Adiabatic HSQC experiments (provided by Bruker as “hsqcetgpsisp2.2”) were conducted using parameters previously defined^40^,^52^. Processing used matched Gaussian apodization in F2 (LB = −0.4, GB = 0.001) and squared cosine-bell apodization in F1 (without linear prediction).

### Enzymatic hydrolysis

Celluclast^®^ 1.5 L was generously provided by Novozymes. The hydrolysis experiments were carried out at 2% (w/v) solids loading in sodium acetate buffer (2 mL, 50 mM, pH 5) swirling at 150 rpm and 50 °C, in a rotary shaker for 72 h, as described previously^53^,^54^. Hydrolysis samples (500 μL) were periodically taken during the course of hydrolysis. The samples were heated at 100 °C for 10 min to inactivate the enzymes and the supernatants were separated and collected after centrifugation at 16,000× *g* for 10 min for the sugar analysis. The concentration of glucose in the supernatants was measured using HPLC (Dionex DX-3000, Sunnyvale, CA) as described elsewhere^29^.

## Additional Information

**How to cite this article**: Singh, R. *et al*. Enhanced delignification of steam-pretreated poplar by a bacterial laccase. *Sci. Rep.* **7**, 42121; doi: 10.1038/srep42121 (2017).

**Publisher's note:** Springer Nature remains neutral with regard to jurisdictional claims in published maps and institutional affiliations.

## Supplementary Material

Supplementary Information

## Figures and Tables

**Table 1:** The list of compounds detected using GC-MS from the soluble portion of SPP incubated with or without sLac.

*t*_R_ (min)	Compound (TMS derivative)
10.5	vanillin^a^ (++)^b^
12.8	4-hydroxybenzoic acid^a^ (+)
14.3	2,6-dimethoxybenzene-1,4-diol (+)
18.8	vanillyl mandelic acid (+)
19.5	palmitic acid (+)
21.4	stearic acid (+)
14.7	syringaldehyde^a^ (−)
14.9	homovanillic acid (−)
15.6	2,3-dihydroxybenzoic acid (−)
15.9	vanillic acid^a^ (−)
16.1	2-(4-hydroxy-3-methoxyphenyl)acetic acid (−)
16.9	protocatechuic acid^a^ (−)
17.9	syringic acid^a^ (−)
18.1	2-(hydroxymethyl)benzene-1,4-diol (−)
23.3–27.6	unidentified polymeric compounds (+/−)

^a^Identity of compound confirmed using authentic standard.

^b^Compound augmented (+) or depleted (−) during incubation with sLac *vs*. control.

**Table 2:** Molar mass distribution of APPL recovered from steam pretreated poplar (SPP) with and without sLac.

	Molar mass distribution				Polydispersity (*M*_w_/*M*_*n*_)	
	Weight average (*M*_*w*_)		Number average (*M*_n_)			
	Peak 1	Peak 2	Peak 1	Peak 2	Peak 1	Peak 2
− sLac	11400 (300)^a^	5900 (200)	9629 (200)	4200 (100)	1.18 (0.04)	1.41 (0.05)
+ sLac	5070 (10)	3320 (10)	4680 (90)	3290 (10)	1.08 (0.04)	1.01 (0.01)

^a^Value in parentheses indicates standard deviation.
