# Supplementary material for: Enhanced delignification of steam-pretreated poplar by a bacterial laccase
Source: Sci Rep. 2017 Feb 7;7:42121. doi: 10.1038/srep42121 (PMC5294454; doi:10.1038/srep42121)
Supplement: Supplementary Information [file srep42121-s1.pdf]

Supplemental Information

Enhanced delignification of steam-pretreated poplar by a bacterial laccase

Rahul Singh[1], Jinguang Hu[2], Matthew R. Regner[3,4], James W. Round[1], John Ralph[3,4], John N. Saddler[2], Lindsay D. Eltis[1,5*]

[1]Department of Microbiology & Immunology, The University of British Columbia, Vancouver, BC, Canada V6T 1Z3

[2]Forest Products Biotechnology/Bioenergy Group, Faculty of Forestry, The University of British Columbia, 2424 Main Mall, Vancouver, BC, V6T 1Z4, Canada.

[3]US Department of Energy Great Lakes Bioenergy Research Center, Wisconsin Energy Institute, Madison, WI 53726, USA

[4]Department of Biochemistry, University of Wisconsin, Madison, WI 53706, USA

[5]Department of Biochemistry and Molecular Biology, The University of British Columbia, Vancouver,

[*]Email: leltis@mail.ubc.ca

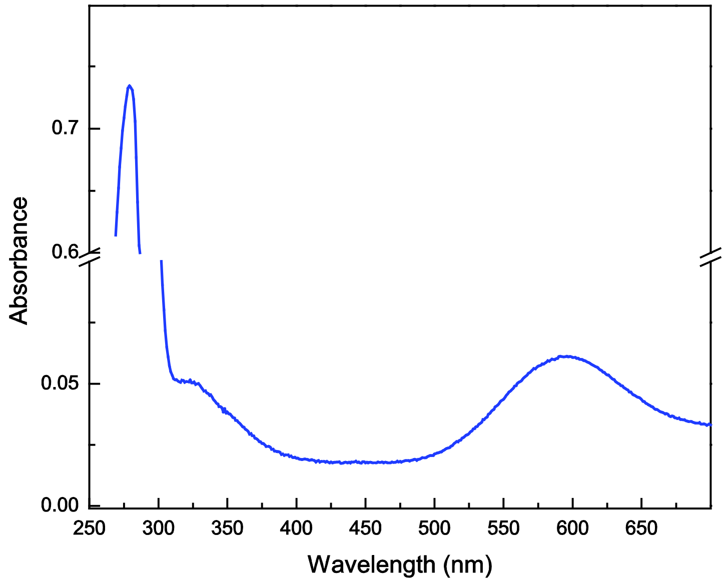

Figure S1. The absorbance spectrum of purified sLac. The sample contained 12 µM sLac in 50 mM Tris-HCl, pH 8 at 25 °C.

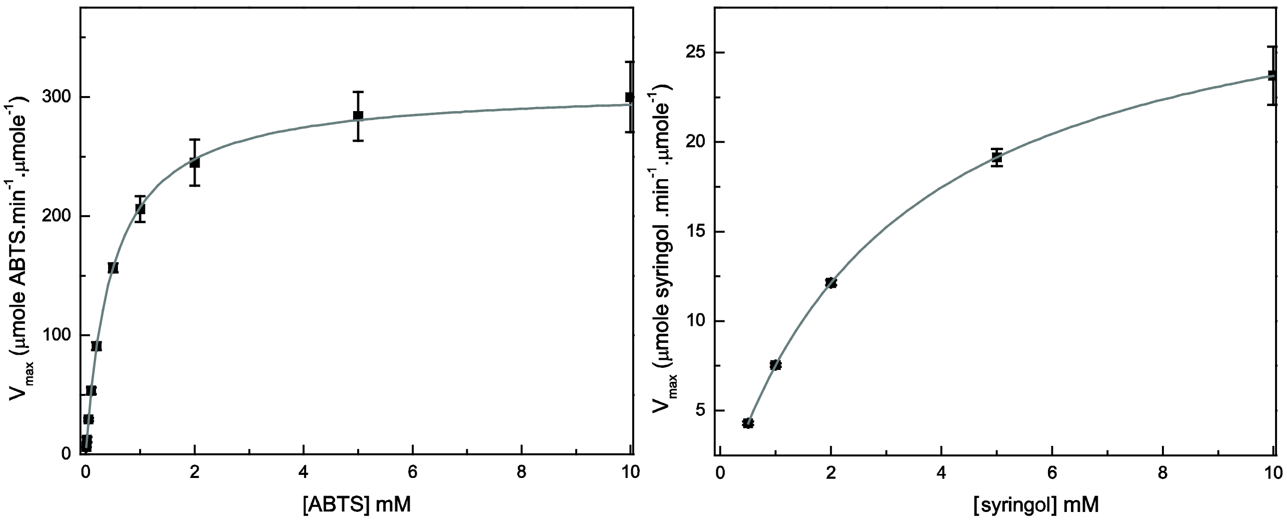

Figure S2. Steady state kinetics analyses of sLac-catalyzed oxidations. The left panel presents the oxidation of ABTS (K_m 0.50 ± 0.04 mM, k_{cat} 7 ± 0.3 s^{-1}; 50 mM sodium acetate, pH 4.5, 30 °C). The right panel presents the oxidation of syringol (K_m 3 ± 0.1 mM; k_{cat} 0.5 ± 0.07 s^{-1}; 50 mM Tris-HCl, pH 8, 30 °C). The curves represent the best fit of the Michaelis-Menten equation to the data using LEONORA.[1]

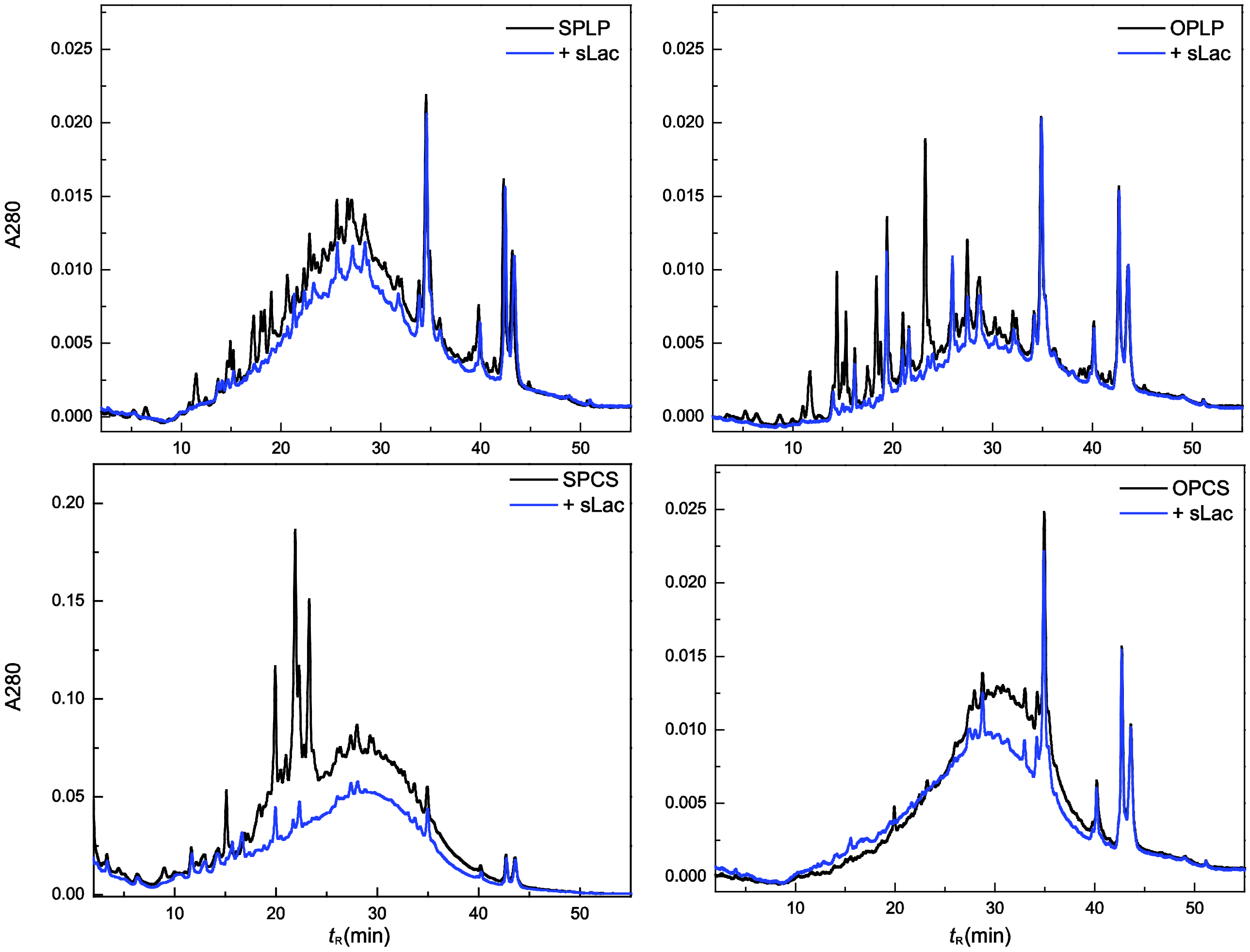

Figure S3. HPLC analyses of the soluble portions of the incubations of steam- (left) or organosolv- (right) pretreated biomass with or without sLac. Reaction mixtures (50 mL) containing 2% (w/v) biomass suspended in 50 mM Tris-HCl, pH 8 with or without sLac were incubated for 48 h at 30 °C and swirled at 200 rpm. The biomass was: lodgepole pine pretreated with steam (SPLP) or organosolv (OPLP); corn stover, pretreated with steam (SPCS) or organosolv (OPCS).

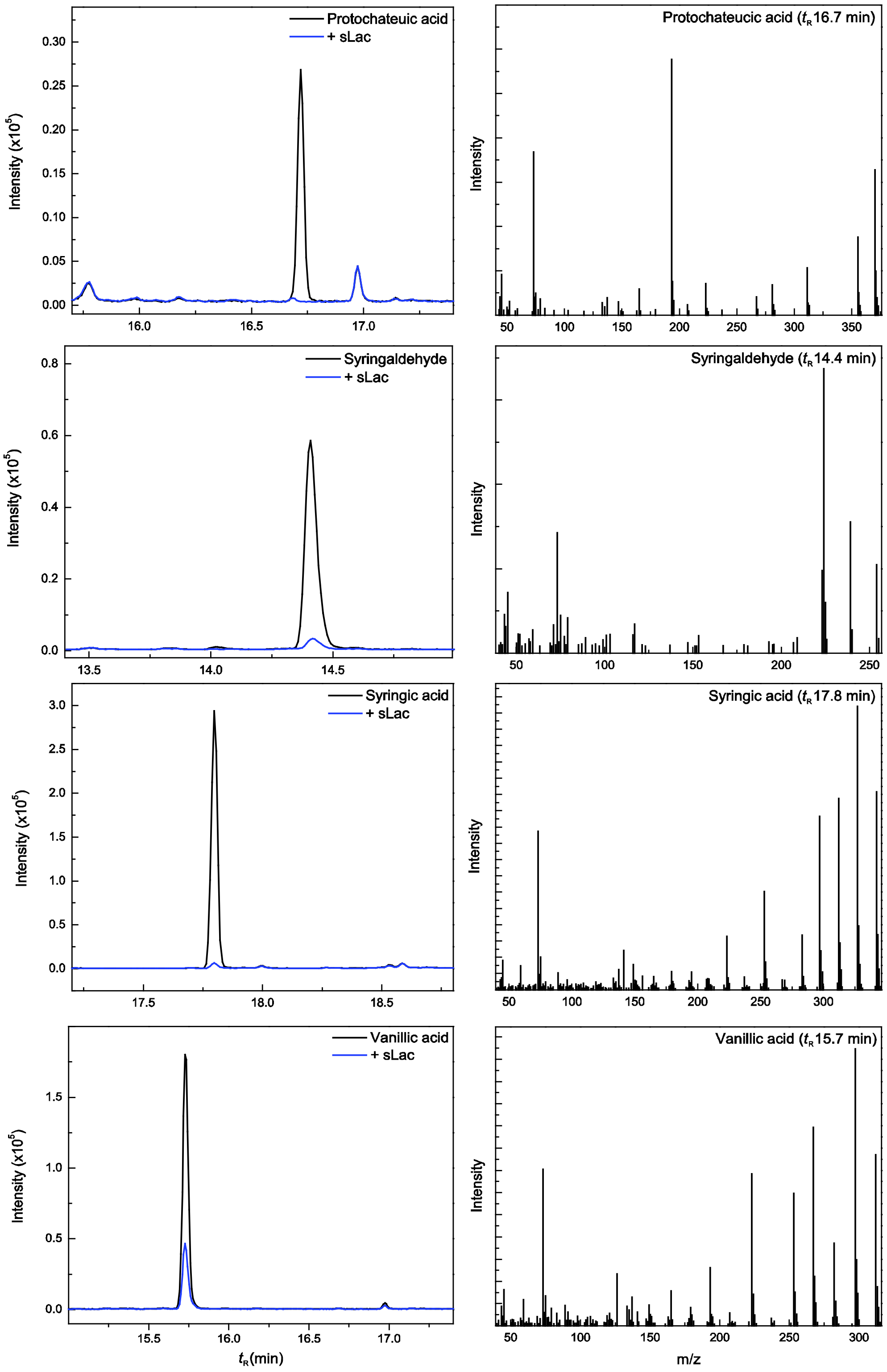

Figure S4. GC-MS analyses of the sLac-catalyzed oxidation of select monoaryls. The left panels show gas chromatograms of the TMS-derivatized extracts of samples described in Figure 3. Black traces represent no-enzyme controls. The right panels shows the associated mass-spectra.

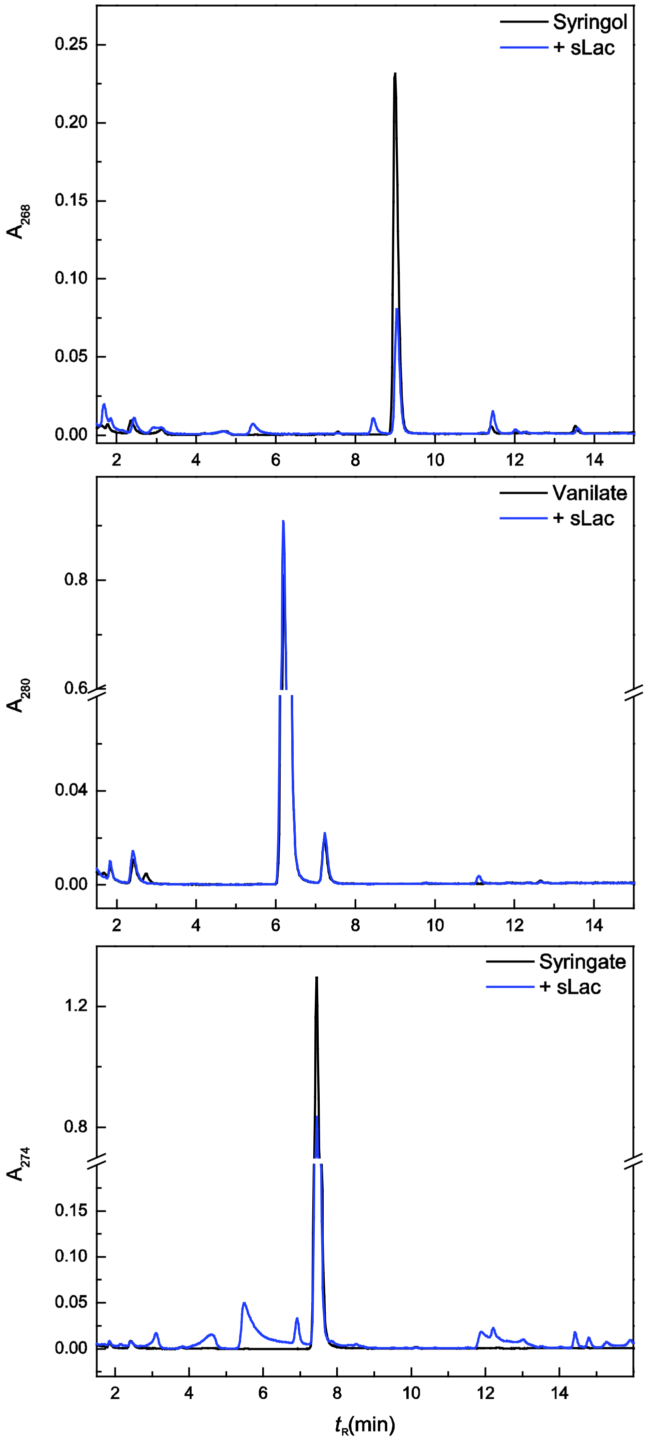

Figure S5. Comparison of sLac catalyzed oxidation of syringol (top), syringate (middle) and vanillate (bottom). The samples were incubated with or without sLac for 2 h at 30 °C and swirled at 200 rpm. Post incubation, samples were acidified and filtered before injection over the HPLC column.

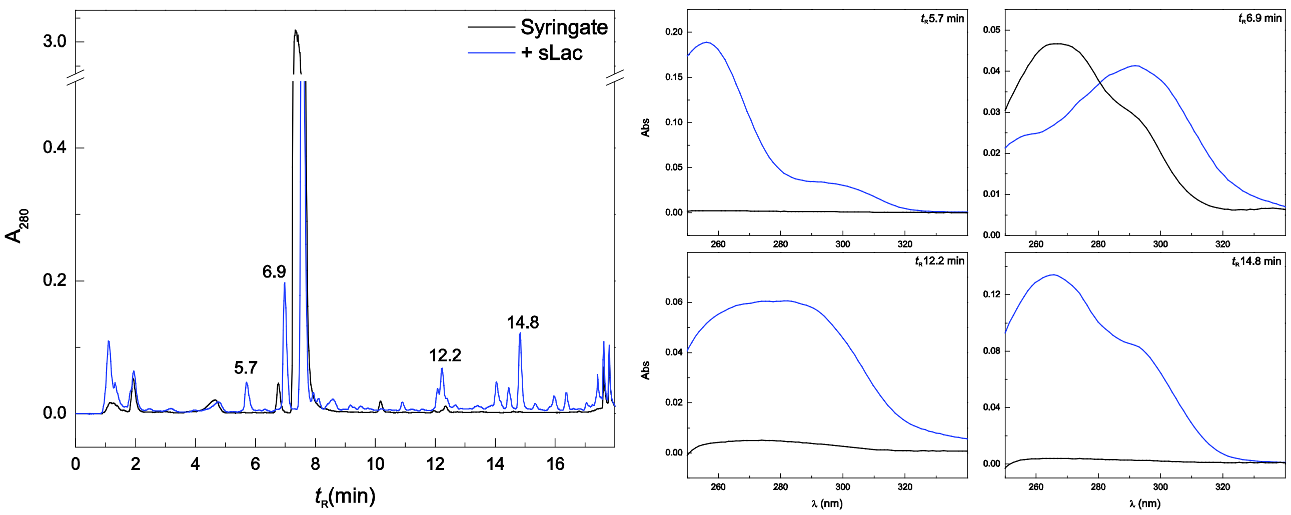

Figure S6. HPLC elution profile (left) of the extracts from reaction mixtures containing syringate incubated with or without sLac. The panel on the right shows absorption spectra of selected peaks that appeared during incubation.

Table S1. Klason lignin analysis of SPP treated with or without sLac.

Sample	Acid-insoluble lignin[a]	Acid-soluble lignin	Total lignin
SPP	28 (1)[b]	1.38 (0.03)	30 (1)
+sLac	28 (1)	1.17 (0.01)	29 (1)

[a]The yield of lignin is reported as percentage of the starting mass. [b]The value in parentheses indicates standard deviation.

References

1. A, Cornish.-Bowden. *Analysis of enzyme kinetic data. New York: Oxford University Press.* (Oxford University Press, 1995).
